# Methylation of *SFRP2* gene as a promising noninvasive biomarker using feces in colorectal cancer diagnosis: a systematic meta-analysis

**DOI:** 10.1038/srep33339

**Published:** 2016-09-23

**Authors:** Qihua Yang, Tao Huang, Guoliang Ye, Bojun Wang, Xinjun Zhang

**Affiliations:** 1 The Affiliated Hospital of Ningbo University, Ningbo, Zhejiang, 315020, China

## Abstract

Methylation of secreted frizzled-related protein genes (SFRP) associated with the Wnt signaling pathway has previously been reported. However, the diagnostic role of SFRP methylation in colorectal cancer (CRC) remains unclear. A systematic search was performed to identify eligible articles for analysis. The pooled OR showed that *SFRP1*, *SFRP2*, *SFRP4* and *SFRP5* methylation was significantly higher in CRC and benign mucosal lesions than in normal colonic mucosa. When CRC was compared to benign mucosal lesions, *SFRP1* and *SFRP2* methylation had a significantly higher OR, but methylated *SFRP4* and *SFRP5* had a similar OR. Moreover, the pooled sensitivity, specificity and AUC (area under the curve) of methylated *SFRP2* in feces of patients with CRC vs. healthy subjects was 0.71, 0.94 and 0.94, respectively. Therefore, methylation of *SFRP1* and *SFRP2* may be significantly correlated with CRC. However, in a study with small sample size, methylated *SFRP4* and *SFRP5* were not shown to be closely associated with CRC. Additionally, detection of *SFRP2* methylation in feces presents a potential noninvasive biomarker for CRC diagnosis.

Colorectal cancer (CRC) is the third most common cancer and the fourth leading cause of mortality of all cancers worldwide^1^. Based on global cancer statistics, approximately 1.4 million new cases were diagnosed in clinics, and approximately 693,900 deaths occurred due to CRC in 2012^2^. Although colonoscopy is the gold standard in the detection of CRC, it can miss adenomas and cancers^2^. Histopathological examination is the most accurate means of diagnosing CRC, and most postoperative patients have a five-year relative survival rate ranging from 50% to 90%^3^. It is necessary to provide promising noninvasive biomarkers with better sensitivity and specificity for the diagnosis of CRC in clinics.

Epigenetic silencing of tumor suppressor genes is an important mechanism in colorectal oncogenesis. Based on the advantages of DNA methylation, such as the convenience of detection, low cost, stability and non-invasive nature of methylation as a diagnostic biomarker, it could become a powerful biomarker for the early detection and diagnosis of colorectal cancer^4^. DNA methylation has previously been used as a biomarker in the detection of human diseases^5^,^6^.

The secreted frizzled-related proteins (SFRPs) are associated with Frizzled (Fz) proteins as a family of soluble proteins. SFRPs can bind to Wnt proteins as antagonists to inhibit signal activation^7^. Inactivation of SFRP genes leads to deregulated activation of Wnt signaling, which is related to CRC^8^. At present, *SFRP2* promoter methylation in feces can be a noninvasive biomarker for the diagnosis of CRC^9^,^10^. *SFRP1* promoter methylation can also be used as a noninvasive tool in CRC diagnosis^11^,^12^.

Although the association of methylated *SFRP1* and *SFRP2* has been investigated in patients with CRC in a meta-analysis^13^,^14^, these methylation studies were conducted using a small sample size. In our study, we first determined whether *SFRP1* and *SFRP2* methylation was associated with CRC in feces and blood. Moreover, the relationship between the methylation of *SFRP4* or *SFRP5* and CRC was also first assessed. Thus, we conducted a meta-analysis for all available studies to evaluate the relationships between *SFRP* gene methylation and CRC based on a large sample size. The pooled sensitivity, specificity and AUC were conducted to assess the diagnostic value of methylated *SFRP2* as a potential non-invasive biomarker in feces.

## Results

### Included study characteristics

**Figure 1:**
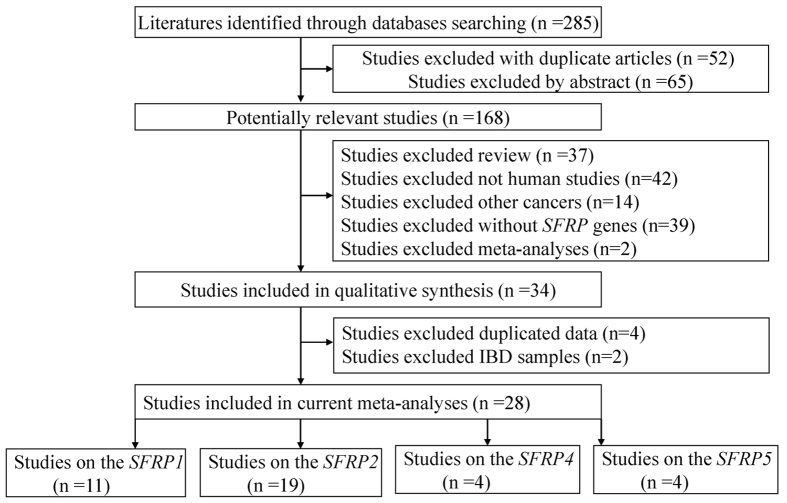
Flow chart of the selection procedure in this study.

Initially, a total of 285 potentially relevant articles were identified from PubMed, EMBASE, Cochrane Library, Web of Science, ProQuest, CNKI and China Wan fang literature databases. The search procedure is shown in Fig. 1, and eligible datasets were included on the basis of the defined inclusion criteria. Finally, 28 eligible case-control studies were included in the current meta-analyses. These included 19 studies on *SFRP2* methylation^5^,^9^,^10^,^15^,^16^,^17^,^18^,^19^,^20^,^21^,^22^,^23^,^24^,^25^,^26^,^27^,^28^,^29^,^30^, 11 studies on *SFRP1* methylation^11^,^12^,^22^,^26^,^28^,^31^,^32^,^33^,^34^,^35^,^36^, 4 studies on *SFRP4* methylation^15^,^22^,^26^,^37^, and 4 studies on *SFRP5* methylation^15^,^22^,^26^,^37^. The information for *SFRP1*, *SFRP2*, *SFRP4* and *SFRP5* methylation was collected from eligible studies and was shown in Table S1.

### Associations of *SFRP1*, *SFRP2*, *SFRP4* and *SFRP5* methylation

**Figure 2:**
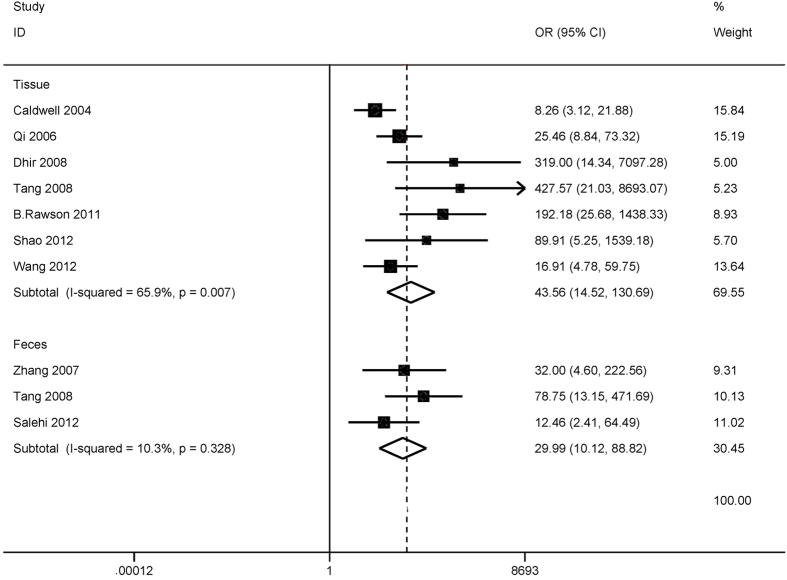
Forest plot for the association with methylated *SFRP1* showing the pooled OR from 7 studies of tissue with 403 CRC and 296 normal colonic mucosa and 3 fecal studies with 83 CRC and 59 healthy subjects; OR = 43.56, P < 0.001 and OR = 29.99, P < 0.001, respectively. Abbreviations: CI, confidence interval; OR, odds ratio; CRC, colorectal cancer.

**Figure 3:**
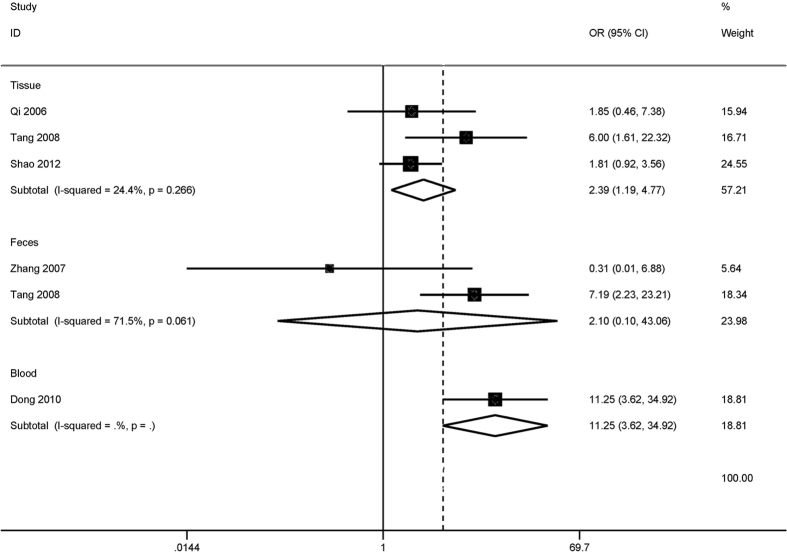
Forest plot for the association of *SFRP1* methylation status showing the pooled OR from 3 studies of tissue with 201 CRC and 144 benign mucosal lesions, 2 studies of feces with 58 CRC and 58 benign mucosal lesions, and 1 study of blood with 72 CRC and 40 benign mucosal lesions; OR = 2.39, P = 0.014, OR = 2.10, P = 0.631, and OR = 11.25, P < 0.001, respectively. Abbreviations: CI, confidence interval; OR, odds ratio; CRC, colorectal cancer.

**Figure 4:**
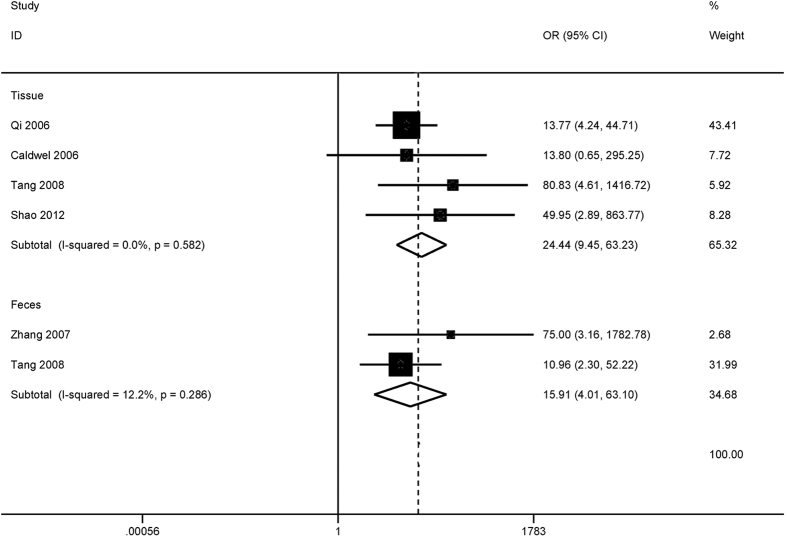
Forest plot for the association of *SFRP1* methylation status showing the pooled OR from 4 studies of tissue involving 155 benign mucosal lesions and 109 normal colonic mucosa and 2 studies of feces involving 58 benign mucosal lesions and 34 healthy subjects; OR = 24.44, P < 0.001 and OR = 15.91, P < 0.001, respectively. Abbreviations: CI, confidence interval; OR, odds ratio; CRC, colorectal cancer.

**Figure 5:**
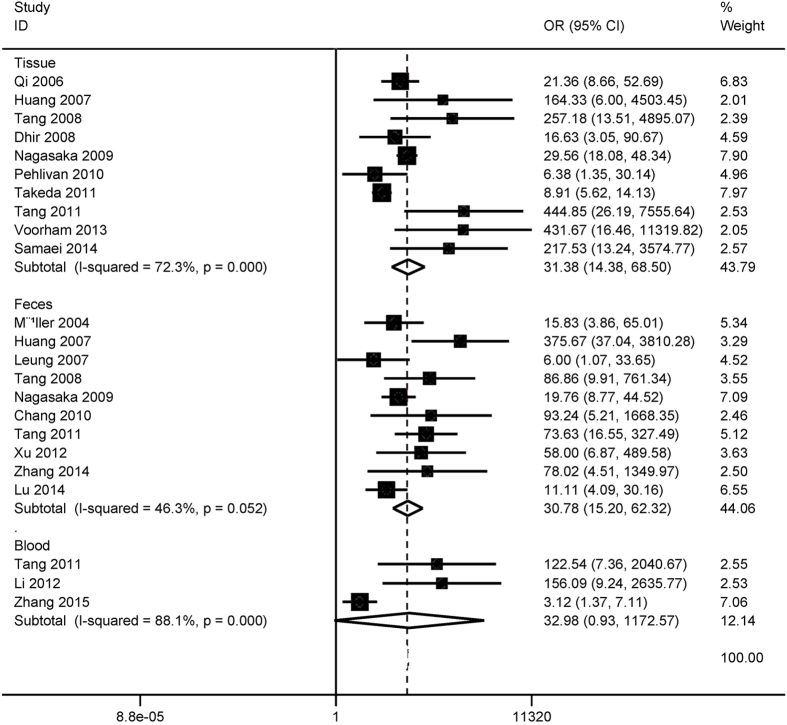
Forest plot for the correlation of methylated *SFRP2* showing the pooled OR from 10 studies of tissue including 936 CRC vs 794 normal colonic mucosa, 10 studies of feces including 551 CRC vs 127 normal healthy feces, and 3 studies of blood samples, including 295 CRC vs 390 healthy blood samples; OR = 31.38, P < 0.001, OR = 30.78, P < 0.001, and OR = 32.98, P = 0.055, respectively. Abbreviations: CI, confidence interval; OR, odds ratio; CRC, colorectal cancer.

**Figure 6:**
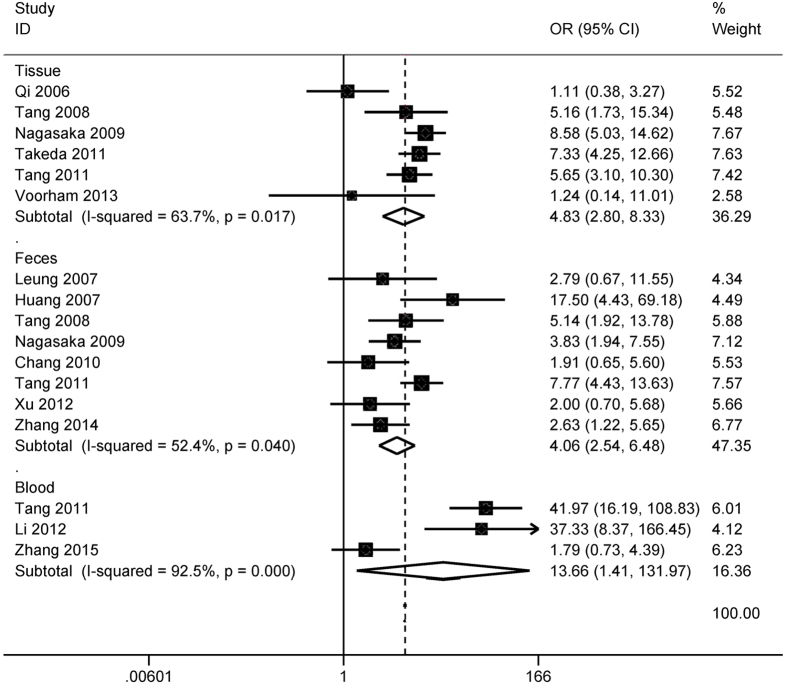
Forest plot for the correlation of *SFRP2* methylation status showing the pooled OR from 6 studies of tissue including 763 CRC vs 487 benign mucosal lesions, 8 studies of feces samples, including 472 CRC vs 409 benign mucosal lesions, and 3 studies of blood samples, including 295 CRC vs 189 benign mucosal lesions; OR = 4.83, P < 0.001, OR = 4.06, P < 0.001, and OR = 13.66, P = 0.024, respectively. Abbreviations: CI, confidence interval; OR, odds ratio; CRC, colorectal cancer.

**Figure 7:**
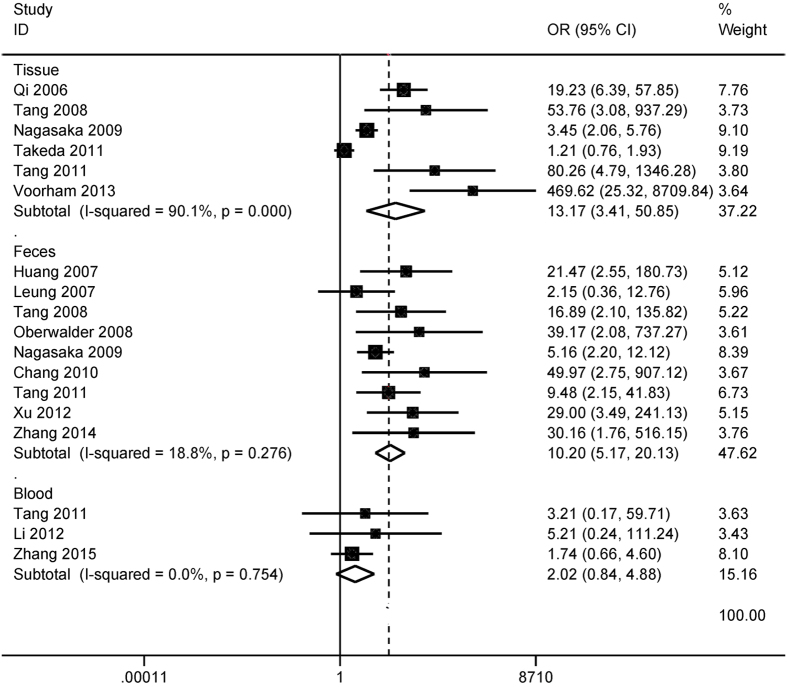
Forest plot of the correlation of *SFRP2* methylation status, showing the pooled OR from 6 studies of tissue samples involving 487 benign mucosal lesions vs 614 normal colonic mucosa, 9 studies of feces samples involving 428 benign mucosal lesions vs 334 normal healthy feces, and 3 studies of blood samples involving 189 benign mucosal lesions vs 127 normal healthy blood samples. OR = 13.17, P < 0.001, OR = 10.20, P < 0.001, and OR = 2.02, P = 0.119, respectively. Abbreviations: CI, confidence interval; OR, odds ratio; CRC, colorectal cancer.

In the current study, significant heterogeneity was performed using the random effects model to make the results more reliable (I^2^ > 50%). Forest plots of the different sample types for the association of methylated *SFRP1* and *SFRP2* were listed in Figs 2, 3, 4, 5, 6 and 7. Due to a very small sample size (CRC = 228), no association between methylated *SFRP4* and *SFRP5* and CRC was observed in our study (Figure S1).

### Association of *SFRP1* methylation in tissue

Our results showed that the overall OR of *SFRP1* methylation in CRC was significantly higher than that in normal colonic mucosa and benign mucosal lesions (OR = 43.56, P < 0.001 and OR = 2.39, P = 0.014, respectively). These data included 7 studies of 403 CRC patients vs 296 normal colonic mucosa and 3 studies of 201 CRC patients vs 144 benign mucosal lesions (Figs 2 and 3). In addition, the pooled OR from 4 studies of 155 benign mucosal lesions vs 109 normal colonic mucosa showed that *SFRP1* methylation in benign mucosal lesions was significantly higher than in normal colonic mucosa (OR = 24.44 and P < 0.001) (Fig. 4). The pooled OR from the comparison of normal colonic mucosa, benign mucosal lesions, and CRC showed that *SFRP1* methylation was significantly associated with CRC.

### Association of *SFRP2* methylation in tissue

The pooled *SFRP2* methylation analysis included 10 studies and 6 studies involved in the comparison of 936 CRC patients and 794 normal colonic mucosa and the comparison of 763 CRC patients and 487 benign mucosal lesions. Our results demonstrated that *SFRP2* methylation in CRC had a higher OR than in normal colonic mucosa and benign mucosal lesions (OR = 31.38, P < 0.001 and OR = 4.83, P < 0.001, respectively) (Figs 5 and 6). Moreover, the pooled OR from 6 studies including 487 benign mucosal lesions and 614 normal colonic mucosa showed that *SFRP1* methylation in benign mucosal lesions was significantly higher than in normal colonic mucosa (OR = 13.17 and P < 0.001) (Fig. 7). Our result demonstrated that *SFRP2* methylation was significantly correlated with CRC.

### Association of *SFRP4* and *SFRP5* methylation in tissue

In the comparison of 228 CRC patients and 225 normal colonic mucosa a meta-analysis involving 4 studies indicated that *SFRP4* methylation was significantly higher in CRC than in normal colonic mucosa (OR = 58.72, P < 0.001). In addition, a significant relationship from 2 studies including 63 benign mucosal lesions and 73 normal colonic mucosa was observed between *SFRP4* methylation and benign mucosal lesions (OR = 12.50, P = 0.005). However, *SFRP4* methylation from 2 studies had a similar OR in 87 CRC patients and 63 benign mucosal lesions (P = 0.06) (Figure S1). Our result showed that *SFRP4* methylation was not correlated with CRC.

A meta-analysis of 4 studies with 228 CRC patients and 225 normal colonic mucosa revealed that the methylation of *SFRP5* in CRC was significantly higher than in normal colonic mucosa (OR = 19.12, P < 0.001). In addition, a significant association from 2 studies including 63 benign mucosal lesions and 73 normal colonic mucosa was found between *SFRP5* methylation and benign mucosal lesions (OR = 23.50 and P = 0.01). While no significant association was found in the comparison of 87 CRC patients and 63 benign mucosal lesions (P = 0.183) (Figure S1). We found that *SFRP5* methylation was not associated with CRC. However, the results should be evaluated carefully, as only two studies with small sample sizes were included. Therefore, it is necessary to further validate the result of *SFRP4* and *SFRP5* methylation based on larger sample sizes in the future.

### Subgroup analyses in tissue

Subgroup analyses of *SFRP1* and *SFRP2* methylation status with significant heterogeneity were conducted to find the differences in detection methods based on ethnicity (Caucasians and Asians), and methylation detection methods (MSP and non-MSP) in tissue (Table 1).

### Impact of ethnicity in tissue

The study population consisted of Caucasian and Asian patients (Table 1). When CRC was compared to normal colonic mucosa, a subgroup ethnicity analysis showed that there was significant association between methylated *SFRP1* and CRC in Asians and Caucasians (OR = 34.44, P < 0.001 and OR = 64.75, P = 0.004, respectively). Significant correlation was also observed between methylated *SFRP2* and CRC in Asians and Caucasians (OR = 37.86, P < 0.001 and OR = 22.49, P = 0.001, respectively). Our results suggested that methylated *SFRP1* or *SFRP2* was associated with Asian and Caucasian populations with CRC.

### Impact of method in tissue

In the current study, the non-MSP detection method primarily included QMSP (quantitative methylation-specific PCR) and COBRA (Combined Bisulfite Restriction Analysis) (Table 1). When CRC was compared to normal colonic mucosa, the OR of methylated *SFRP2* for the MSP subgroup was 69.12 (95% CI: 18.78–254.42), and for the non-MSP subgroup the OR was 17.37 (95% CI: 6.14–49.12). In comparing CRC and benign lesions, the OR of methylated *SFRP2* for the MSP subgroup was 3.38 (95% CI: 1.27–8.96), and the OR for the non-MSP subgroup was 7.24 (95% CI: 4.40–11.91). In comparing the benign mucosal lesions and normal colonic mucosa, the OR of methylated *SFRP2* for the MSP subgroup was 25.57 (95% CI: 9.73–67.16), and the OR for the non-MSP subgroup was 4.88 (95% CI: 1.13–21.09). Our findings suggested that methylated *SFRP2* was correlated with CRC in the MSP and non-MSP detection methods in tissue.

### Impact of heterogeneity on results in tissue

We assessed the differences in heterogeneity for the methylated *SFRP1* and *SFRP2* based on subgroup analyses, as shown in Table 1. When CRC was compared to normal colonic mucosa, the subgroup analyses based on ethnicity showed that *SFRP1* methylation had a significant heterogeneity in the Caucasians subgroup (I^2^ = 84.4%) but not in the Asian subgroup (I^2^ = 35.9%), which suggests that the heterogeneity was from the Caucasian population. Subgroup analyses based on ethnicity demonstrated that *SFRP2* methylation had strong heterogeneity in both the Caucasian and Asian subgroups (I^2^ = 62.7% and I^2^ = 77.8%, respectively). In addition, subgroup analyses of detection methods revealed that *SFRP2* methylation had strong heterogeneity in the MSP and non-MSP subgroups (I^2^ = 54.7% and I^2^ = 82.8%, respectively). Our findings suggested that ethnicity and detection methods were not the sources of heterogeneity.

In the comparison of the CRC and benign mucosal lesions, subgroup analyses of *SFRP2* methylation based on detection methods showed that the MSP subgroup had a larger heterogeneity (I^2^ = 70.9%) than the non-MSP subgroup (I^2^ = 30.2%), suggesting that MSP subgroup was a main source of heterogeneity. According to detection methods from the comparison of benign mucosal lesions and normal colonic mucosa, significant heterogeneity of *SFRP2* methylation was found in the non-MSP subgroup (I^2^ = 91.4%), but not in the MSP subgroup (I^2^ = 0.0%). The results suggested that the heterogeneity was from the non-MSP subgroup.

### Pooled OR of *SFRP1* methylation in feces and blood

We first determined whether *SFRP1* and *SFRP2* methylation was associated with CRC in blood and feces. The pooled OR from 3 studies with 83 CRC and 59 normal healthy feces samples showed that methylated *SFRP1* in CRC was significantly higher than in normal healthy feces (OR = 29.99, P < 0.001) (Fig. 2). The pooled OR from 2 studies involving 58 benign mucosal lesions feces samples and 34 normal healthy feces samples showed that methylated *SFRP1* in benign mucosal lesions was significantly higher than in normal healthy feces (OR = 15.91, P < 0.001) (Fig. 4). However, *SFRP1* methylation in 2 studies with 58 CRC and 58 benign mucosal lesions had a similar OR in the feces of patients with CRC and benign mucosal lesions (P = 0.631) (Fig. 3). Our findings suggested that *SFRP1* methylation was not correlated with CRC in feces. Only 1 study of blood with 72 CRC and 40 benign mucosal lesions showed that *SFRP1* methylation had a higher OR in blood samples from patients with CRC than in blood samples from patients with benign mucosal lesions (OR = 11.25, P < 0.001) (Fig. 3). However, the results should be interpreted with caution based on the small sample size. More studies based on feces and blood samples are very essential to further evaluate the association of *SFRP1* methylation in feces and blood.

### Pooled OR of *SFRP2* methylation in blood samples

The pooled OR from 3 studies involving blood samples from 295 patients with CRC and 127 healthy subjects showed that methylated *SFRP2* had a similar OR in CRC and healthy subjects (P = 0.055) (Fig. 5). The pooled OR from 3 studies involving blood samples from 295 patients with CRC and 189 patients with benign mucosal lesions showed that methylated *SFRP2* in CRC was significantly higher than in benign mucosal lesions (OR = 13.66, P = 0.024) (Fig. 6). In addition, *SFRP2* methylation from 3 studies of 189 benign mucosal lesions and 127 healthy blood subjects showed a similar OR in benign mucosal lesions and healthy blood subjects (P = 0.119) (Fig. 7). Our results suggested that *SFRP2* methylation was not correlated with CRC in blood samples. However, the results should be interpreted carefully because of the small sample size. More studies of blood samples are necessary to further evaluate the correlation of *SFRP2* methylation.

### Pooled OR of *SFRP2* methylation in feces

The results from 10 studies of 551 CRC and 390 healthy subjects and 8 studies of 472 CRC and 409 benign mucosal lesions patients showed that *SFRP2* methylation in feces from patients with CRC had a higher OR than in the feces of healthy subjects and patients with benign mucosal lesions (OR = 30.78, P < 0.001 and OR = 4.06, P < 0.001, respectively) (Figs 5 and 6). In addition, data from 9 studies on 428 benign mucosal lesion feces samples and 334 healthy feces samples showed that *SFRP2* methylation in the feces of patients with benign mucosal lesions had a higher OR than healthy subjects (OR = 10.20, P < 0.001) (Fig. 7). Our results showed that *SFRP2* methylation was associated with CRC in feces. However, the diagnostic effect with sensitivity, specificity and AUC was unclear; thus, we further calculated the pooled sensitivity, specificity and AUC to evaluate the diagnostic capacity of *SFRP2* methylation for CRC diagnosis in feces.

### Diagnostic capacity of *SFRP2* methylation in feces

**Figure 8:**
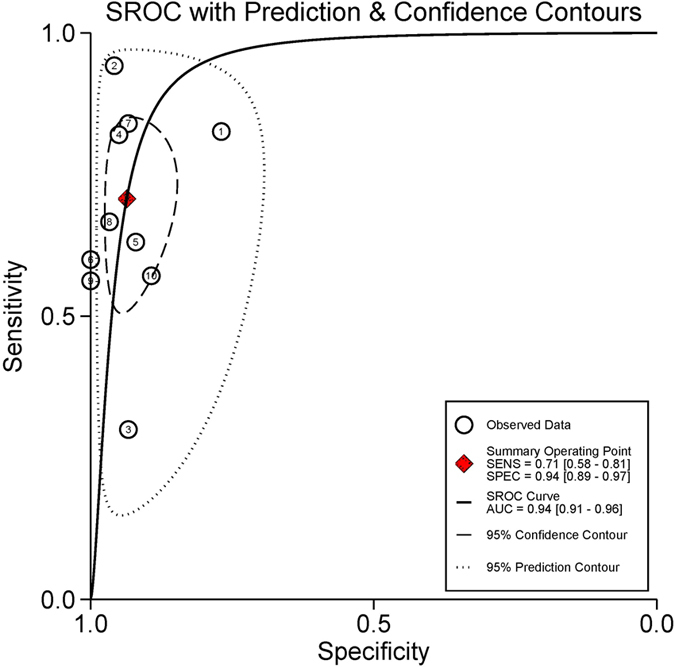
SROC estimation of *SFRP2* methylation detection in feces between CRC patients and healthy subjects, sensitivity = 0.71, specificity = 0.94 and AUC = 0.94. The AUC value was high (AUC = 0.94 > 0.9), which indicated that methylated *SFRP2* can be applied as a promising non-invasive biomarker for CRC diagnosis. The confidence and prediction contours were defined as 95% confidence interval and 95% prediction interval of the pooled sensitivity and specificity in SROC space. Abbreviations: SROC, summary receiver operating characteristics; AUC, the area under the curve of the summary receiver operating characteristic; CRC, colorectal cancer.

The current study was also further performed to assess the diagnostic value of *SFRP2* methylation status as a non-invasive biomarker in feces. In the diagnostic discrimination of CRCs and healthy subjects, the overall sensitivity, specificity and AUC of methylated *SFRP2* were 0.71 (95 CI %: 0.58–0.81), 0.94 (95 CI %: 0.89–0.97) and 0.94 (95 CI %: 0.91–0.96) in feces studies (Fig. 8). The outcome demonstrated that *SFRP2* methylation had a potential diagnostic effect in feces. Based on the use of AUC to assess the accuracy of the diagnostic test (AUC = 0.94 > 0.9), we showed that *SFRP2* methylation had a promising diagnostic effect as a non-invasive biomarker in feces.

**Figure 9:**
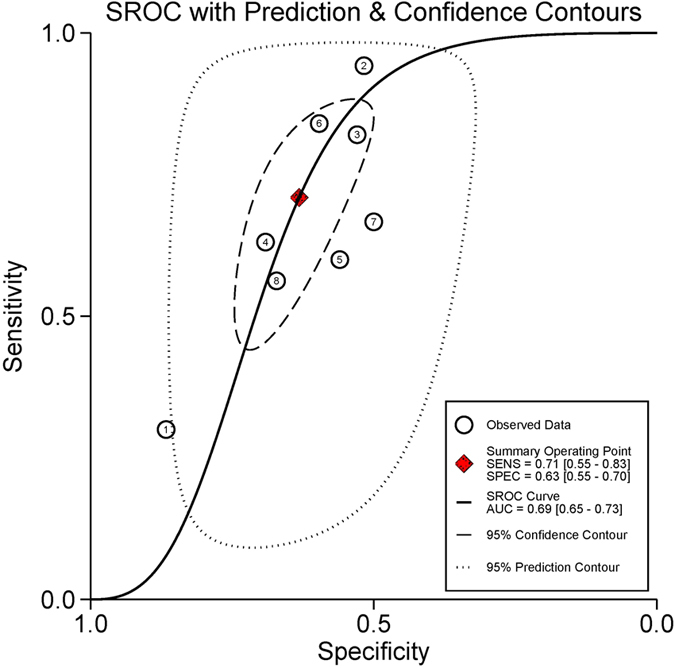
SROC evaluation of *SFRP2* methylation test in feces between CRC patients and benign lesions, sensitivity = 0.71, specificity = 0.63 and AUC = 0.69. The AUC value was low (AUC = 0.69 < 0.7), which indicated that methylated *SFRP2* as a biomarker could not accurately distinguish CRC and benign mucosal lesions. The confidence and prediction contours were defined as 95% confidence interval and 95% prediction interval of the pooled sensitivity and specificity in SROC space. Abbreviations: SROC, summary receiver operating characteristics; AUC, the area under the curve of the summary receiver operating characteristic; CRC, colorectal cancer.

In addition, for the diagnostic discrimination between CRC and benign lesions, the pooled sensitivity, specificity and AUC of methylated *SFRP2* were 0.71 (95 CI %: 0.55–0.83), 0.63 (95 CI %: 0.55–0.70) and 0.69 (95 CI %: 0.65–0.73) in feces samples (Fig. 9), which demonstrated that the use of methylated *SFRP2* as a biomarker could not accurately distinguish CRC and benign lesions. We also found that the mean methylation of the *SFRP2* gene was 0.72 in feces from CRC patients, and 0.38 in feces from benign mucosal lesions patients.

## Discussion

CRC is a prevalent malignancy that develops from normal colon epithelium into either adenomas or serrated polyps, then progresses to cancer via multiple sequences of histological development^22^,^38^. Aberrantly methylated *SFRP* genes have been reported in the pathogenesis of CRC^22^,^26^, but the diagnostic role of gene methylation has not been evaluated in CRC. We performed diagnostic estimations to test the value of gene methylation in CRC diagnosis using feces.

The current study revealed that methylated *SFRP1* and *SFRP2* were significantly associated with CRC. However, significant association was not observed between the *SFRP4* or *SFRP5* methylation status and CRC. The results of methylated *SFRP4* and *SFRP5* should be interpreted with caution as the sample sizes were small, which suggests the need for additional studies with larger sample sizes in the future.

Subgroup analyses were performed to evaluate the impact of ethnicity and testing methods for methylated *SFRP1* and *SFRP2* in tissue. When CRC was compared to normal colonic mucosa, a subgroup analysis of ethnicity suggested that *SFRP1* or *SFRP2* methylation was correlated with Asian and Caucasian populations with CRC. Subgroup analyses based on detection methods revealed that *SFRP2* methylation was associated with CRC in the MSP and non-MSP detection methods. In the current study, normal tissue samples for methylated *SFRP* genes included normal mucosa from healthy individuals and adjacent normal tissue samples (from CRC or adenomas). The adjacent normal samples might have an impure composition that could be slightly contaminated by tumor cells from CRC or adenomas, which could bias the results.

We evaluated the sources of heterogeneity based on subgroup analyses in tissue. When CRC was compared to normal colonic mucosa, subgroup analyses of ethnicity revealed that *SFRP1* methylation exhibited heterogeneity in the Caucasian subgroup. In addition, the outcome of *SFRP1* methylation based on ethnicity and detection methods could not reveal the sources of heterogeneity. When CRC was compared to benign mucosal lesions, subgroup analyses of detection methods suggested that the heterogeneity of *SFRP2* methylation was derived from the MSP subgroup. In the comparison of benign mucosal lesions and normal colonic mucosa, we found that the heterogeneity of *SFRP2* methylation was derived from the non-MSP subgroup.

We also analyzed the pooled OR of methylated *SFRP1* and *SFRP2* to determine whether these two genes were associated with CRC in feces and blood. Finally, our results suggested that methylated *SFRP2* was correlated with CRC in feces. Moreover, some studies suggest that the detection of DNA methylation from feces can be used as a diagnostic tool in patients with CRC^9^,^24^,^27^. Therefore, we further evaluated the diagnostic effect of methylated *SFRP2* in feces. Based on benign mucosal lesions as the control standard, the outcome showed that methylated *SFRP2* could not accurately distinguish CRC and benign disease in feces (specificity = 0.63 and AUC = 0.69). Based on normal healthy samples as a control standard, the pooled sensitivity, specificity and AUC of methylated *SFRP2* in the current study were 0.71, 0.94 and 0.94, respectively. We found that the AUC of *SFRP2* methylation status in feces (AUC = 0.94 > 0.9) was very large. In addition, the mean methylation of the *SFRP2* gene was significantly higher in CRC than in benign mucosal lesions (0.72 vs 0.38), which indicated that detection of *SFRP2* methylation could be a promising noninvasive biomarker for the diagnosis of CRC using feces.

Compared with the previous meta-analysis of *SFRP1* methylation status^14^, the number of studies included in the present study (n = 11) was greater than in the previous study (n = 8). The previous study also only analyzed *SFRP1* methylation in tissue samples, but our study included other sample types (blood or feces), thus, we determined whether *SFRP1* methylation was correlated with CRC in feces. Finally, the previous study did not analyze the correlation of *SFRP1* methylation in the comparison of benign mucosal lesions and normal colonic mucosa. Moreover, in comparison with the previous meta-analyses on *SFRP2* methylation^13^, our meta-analyses included 19 studies, which was 5 more than the previous study. Our meta-analyses further studied the OR of *SFRP2* methylation between cancer samples and benign mucosal lesions, which the previous meta-analyses did not. In addition, we determined whether *SFRP2* methylation was associated with CRC in feces and blood. Because the diagnostic effect of methylated *SFRP2* remains unclear in CRC, the present study integrated sensitivity, specificity and AUC measures to evaluate the diagnostic role of methylated *SFRP2*.

There were some limitations in our study. First, although we tried to minimize selection bias using PubMed, EMBASE, Cochrane Library, Web of Science, ProQuest, CNKI and China Wan fang literature databases, studies with other languages and other styles such as conference abstracts could have been missed, which might introduce selection bias because the studies were restricted to articles published in English or Chinese. Second, the main ethnic populations in our study were Asians and Caucasians, and other ethnic groups, such as Africans, were absent. Third, the studies using blood samples were relatively small. Therefore, the sensitivity, specificity and AUC of methylated *SFRP* genes in blood samples were not calculated. Blood samples were also very important because of their ease of collection in the clinic, and might be promising for identifying biomarkers for CRC diagnosis. Therefore, more studies will be essential to validate the diagnostic capacity of methylated *SFRP* genes in blood samples in the future. Finally, in our study, we found that methylated *SFRP4* and *SFRP5* were not closely associated with CRC, possibly due to the smaller sample size (CRC = 228), which suggests that more studies are necessary to validate our results in the future.

In conclusion, the current study provides strong evidence that methylated *SFRP1* and *SFRP2* may be significantly correlated with CRC, while methylated *SFRP4* and *SFRP5* may not be associated with CRC. Moreover, *SFRP*2 methylation has a potential diagnostic effect in feces and can be applied as a non-invasive biomarker in the clinical diagnosis of CRC. However, due to the very small sample size of methylated *SFRP4* and *SFRP5* in the current study, additional studies will be needed to validate the results based on larger sample sizes in the future.

## Materials and Methods

### Literature search

A systematic search was performed to identify the relevant literature from PubMed, EMBASE, Cochrane Library, Web of Science, ProQuest, CNKI and China Wanfang literature databases through June 2015, without language restrictions. We used the following keywords and search terms: (Wnt OR frizzled related protein OR SFRP OR secreted frizzled-related protein) and (colorectal cancer OR colorectal tumor OR colorectal carcinoma OR colorectal neoplasm) AND (methylation OR epigene*).

### Selection criteria of studies included in the meta-analysis

The following inclusion criteria were used to identify eligible studies: (1) the study was a case-control study detailing the methylation detection methodology, including tissue, blood or fecal samples; (2) sufficient data could be assessed to determine the methylation frequency of *SFRP* genes; (3) CRC patients were diagnosed by histopathologic examinations; benign mucosal lesions included adenoma and hyperplastic polyp; and normal colonic mucosa consisted of normal healthy or adjacent normal tissue samples; (4) IBD samples including ulcerative colitis or Crohn’s disease were removed from the analysis; and (5) if authors published more than one paper using the sample data, only the most recent publication with the largest sample size was selected. Literature was excluded if it did not meet the inclusion criteria. The present meta-analysis was reported based on the Preferred Reporting Items for Systematic Reviews and Meta-Analysis (PRISMA) statement (Table S2).

### Data extraction

The following data were extracted from the studies included in the meta-analysis: first author name, clinicopathological histology, type of samples, total number of cases, total number of benign mucosal lesions, total number of normal colonic mucosa samples, detection methods of methylation, and frequency of methylation.

### Statistical analysis

Data were analyzed using STATA software (STATA version 12.0, Stata Corporation, College Station, TX, USA). The pooled odds ratio (OR) with corresponding 95% confidence interval (95% CI) was calculated to evaluate the association between *SFRP* methylation and disease. Heterogeneity was assessed by a chi-square test and Q statistic for case-control studies^39^. The pooled data were calculated using the random effects model. An I^2^ statistic with a value of over 50% represented significant heterogeneity, and subgroup analyses were used to identify potential sources of heterogeneity. Otherwise, the fixed effects model was used, indicating a lack of heterogeneity^40^,^41^. The integrated sensitivity, specificity and AUC (the area under the curve of the summary receiver operating characteristic) were calculated to validate the diagnostic effect of *SFRP2* methylation in feces. Although the pooled sensitivity and specificity are two of the most common and important indicators of diagnostic effect, sometimes these measures do not reflect the overall accuracy of the test based on the threshold effect^42^. Because the summary receiver operating characteristics (SROC) analysis indicated the stability and accuracy of the diagnostic detection in the meta-analysis^43^, the AUC was used to assess the accuracy of the *SFRP2* methylation diagnostic test in the current study.

## Additional Information

**How to cite this article**: Yang, Q. *et al*. Methylation of *SFRP2* gene as a promising noninvasive biomarker using feces in colorectal cancer diagnosis: a systematic meta-analysis. *Sci. Rep.* **6**, 33339; doi: 10.1038/srep33339 (2016).

## Supplementary Material

Supplementary Information

## Figures and Tables

**Table 1:** Subgroup analyses for methylated *SFRP1* and *SFRP2* in tissue.

Gene	Tissue/n	Pooled OR (95% CI)	Heterogeneity (I^2^)	P value	Compared group
*SFRP1*	Caucasians/3	64.75 (3.92–1070.36)	84.40%	0.004	CRC vs Normal
	Asians/4	34.44 (11.88–99.83)	35.90%	<0.001	
*SFRP2*	Caucasians/3	22.49 (3.31–152.96)	62.70%	0.001	CRC vs Normal
	Asians/7	37.86 (15.06–95.17)	77.80%	<0.001	
	MSP/6	69.12 (18.78–254.42)	54.70%	<0.001	
	Non-MSP/4	17.37 (6.14–49.12)	82.80%	<0.001	
*SFRP2*	MSP/3	3.38 (1.27–8.96)	70.90%	0.015	CRC vs Benign
	Non-MSP/3	7.24 (4.40–11.91)	30.20%	<0.001	
*SFRP2*	MSP/3	25.57 (9.73–67.16)	0.00%	<0.001	Benign vs Normal
	Non-MSP/3	4.88 (1.13–21.09)	91.40%	0.034	

Subgroup analysis based on sample types (tissue, blood or feces), ethnicity (Caucasians and Asians), and methylation detection methods (MSP and Non-MSP) in tissue; MSP: Methylation Specific PCR; n stands for studies; CI: confidence interval; CRC: colorectal cancer; OR: odds ratio; Non-MSP mainly include QMSP (Quantitative methylation-specific PCR) and COBRA (Combined Bisulfite Restriction Analysis); Normal: normal colonic mucosa; Benign: benign mucosal lesions.
