# Supplementary material for: Methylation of SFRP2 gene as a promising noninvasive biomarker using feces in colorectal cancer diagnosis: a systematic meta-analysis
Source: Sci Rep. 2016 Sep 23;6:33339. doi: 10.1038/srep33339 (PMC5034263; doi:10.1038/srep33339)
Supplement: Supplementary Information [file srep33339-s1.pdf]

Supplementary Information

Methylation of *SFRP2* gene as a promising noninvasive biomarker using feces in colorectal cancer diagnosis: a systematic meta-analysis

Qihua Yang [#,*], Tao Huang [#], Guoliang Ye*, Bojun Wang, Xinjun Zhang

The Affiliated Hospital of Ningbo University, Ningbo, Zhejiang, 315020, China

#: Q.H.Y and T.H are co-first authors of this work.

* Corresponding author:

Qihua Yang, the Affiliated Hospital of Ningbo University, Ningbo, Zhejiang, 315020, China.

Phone: +86 13732113594, Email: muzi05@sohu.com

Guoliang Ye, the Affiliated Hospital of Ningbo University, Ningbo, Zhejiang, 315020, China.

Phone: +86 13606788000, Email: ndfyygl@163.com

Table S1: The main characteristics of all available studies.

First author	Ethnicity	Method	Sample	Cancer		Benign		Normal		Gene
				M+	Total	M+	Total	M+	Total	
Caldwell 2004	Caucasians	MSP/COBRA	Tissue	40	51			11	36	*SFRP1*
Qi 2006	Asains	MSP	Tissue	67	72	29	33	20	58	*SFRP1*
Caldwel 2006	Caucasians	COBRA	Tissue			11	11	7	11	*SFRP1*
Zhang 2007	Caucasians	MSP	Feces	16	19	7	7	2	14	*SFRP1*
Tang 2008	Asains	MSP	Tissue	36	39	34	51	0	20	*SFRP1*
Tang 2008	Asains	MSP	Feces	35	39	28	51	2	20	*SFRP1*
Dhir 2008	Caucasians	MSP	Tissue	14	16			0	27	*SFRP1*
Dong 2010	Asains	MSP	Serum	40	72	4	40			*SFRP1*
B.Rawson 2011	Caucasians	Methylight	Tissue	66	100			1	100	*SFRP1*
Salehi 2012	Asains	MSP	Feces	13	25			2	25	*SFRP1*
Wang 2012	Asains	MSP	Tissue	24	35			24	35	*SFRP1*
Shao 2012	Asains	MSP	Tissue	62	90	33	60	0	20	*SFRP1*
Müller 2004	Caucasians	Methylight	Feces	19	23			6	26	*SFRP2*
Qi 2006	Asains	MSP	Tissue	60	72	27	33	11	58	*SFRP2*
Leung 2007	Asains	MSP	Feces	6	20	4	30	2	30	*SFRP2*
Huang 2007	Asains	MSP	Feces	49	52	14	29	1	24	*SFRP2*
Huang 2007	Asains	MSP	Tissue	14	15			0	8	*SFRP2*
Tang 2008	Asains	MSP	Tissue	34	39	29	51	0	20	*SFRP2*
Tang 2008	Asains	MSP	Feces	32	39	24	51	1	20	*SFRP2*
Dhir 2008	Caucasians	MSP	Tissue	14	16			8	27	*SFRP2*
Oberwalder 2008	Caucasians	Methylight	Feces			8	19	0	26	*SFRP2*
Nagasaka 2009	Asains	COBRA	Feces	53	84	21	68	9	113	SFRP2
Nagasaka 2009	Asains	COBRA	Tissue	209	243	43	103	42	244	SFRP2
Chang 2010	Asains	MSP	Feces	18	30	11	25	0	31	*SFRP2*
Pehlivan 2010	Caucasians	Test[a]	Tissue	9	17			3	20	*SFRP2*
Tang 2011	Asains	MSP	Feces	142	169	44	109	2	30	*SFRP2*
Tang 2011	Asains	MSP	Serum	113	169	5	109	0	30	*SFRP2*
Tang 2011	Asains	MSP	Tissue	149	169	62	109	0	30	*SFRP2*
Takeda 2011	Asains	COBRA	Tissue	192	222	48	103	102	244	*SFRP2*
Xu 2012	Asains	MSP	Feces	20	30	15	30	1	30	*SFRP2*
Li 2012	Asains	MSP	Blood	42	69	2	50	0	50	*SFRP2*
Voorham 2013	Caucasians	QMSP	Tissue	17	18	82	88	0	18	*SFRP2*
Zhang 2014	Asains	MSP	Feces	27	48	22	67	0	30	*SFRP2*
Lu 2014	Asains	MSP	Feces	32	56			6	56	*SFRP2*
Samaei 2014	Asains	MSP	Tissue	58	125			0	125	*SFRP2*
Zhang 2015	Asains	MSP	Plasma	31	57	12	30	13	47	*SFRP2*
Suzuki 2004	Caucasians	MSP	Tissue	6	15	5	30	0	15	*SFRP4*
Qi 2006	Asains	MSP	Tissue	26	72	8	33	1	58	*SFRP4*
Dhir 2008	Caucasians	MSP	Tissue	13	16			0	27	*SFRP4*
Samaei 2014	Asains	MSP	Tissue	38	125			0	125	*SFRP4*
Suzuki 2004	Caucasians	MSP	Tissue	8	15	24	30	0	15	*SFRP5*

Qi 2006	Asains	MSP	Tissue	38	72	19	33	7	58	*SFRP5*
Dhir 2008	Caucasians	MSP	Tissue	10	16			0	27	*SFRP5*
Samaei 2014	Asains	MSP	Tissue	33	125			0	125	*SFRP5*

MSP: Methylation Specific PCR, QMSP: Quantitative methylation-specific PCR, COBRA: Combined Bisulfite Restriction Analysis, Test[a]: The reverse-hybridization method, M+: the number of methylation; Total: the number of case or control.

Figures S1: Forest plots for the association of methylated *SFRP4* and *SFRP5*

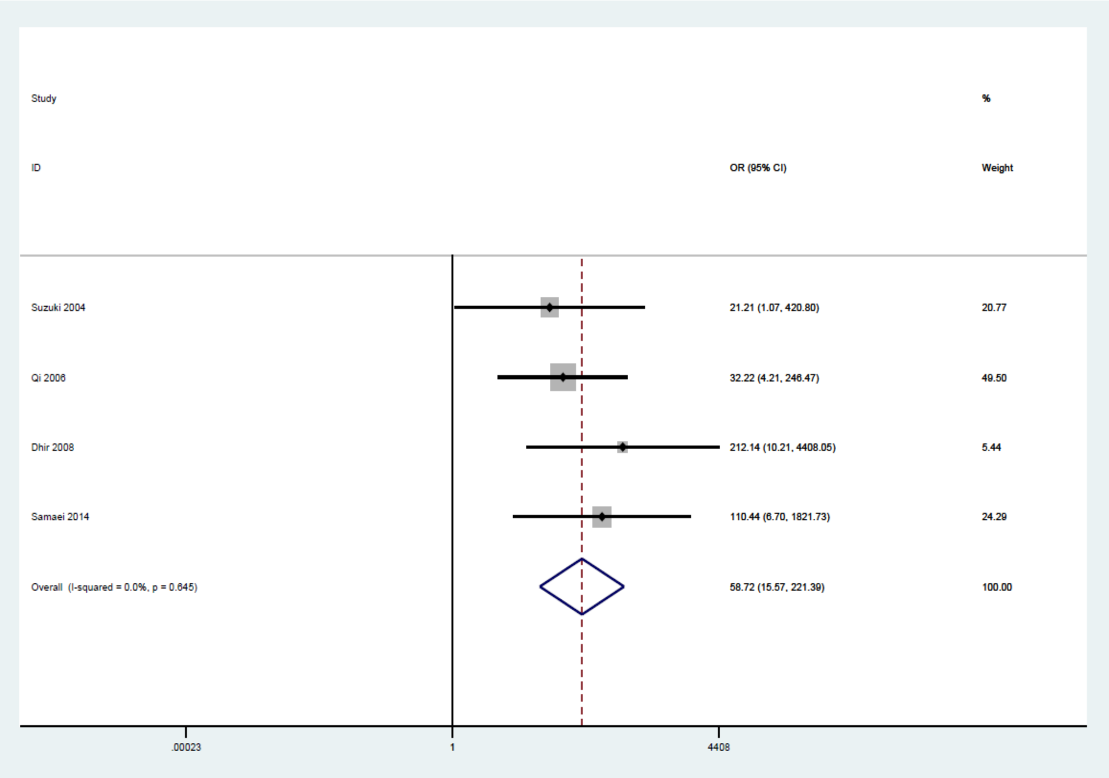

Figure S1: Forest plot for the association of methylated *SFRP4* between 228 CRC patients and 225 normal colonic mucosa, OR = 58.72, 95% CI = 15.57 - 221.39, P < 0.001. Abbreviations: CI, confidence interval; OR, odds ratio; CRC, colorectal cancer.

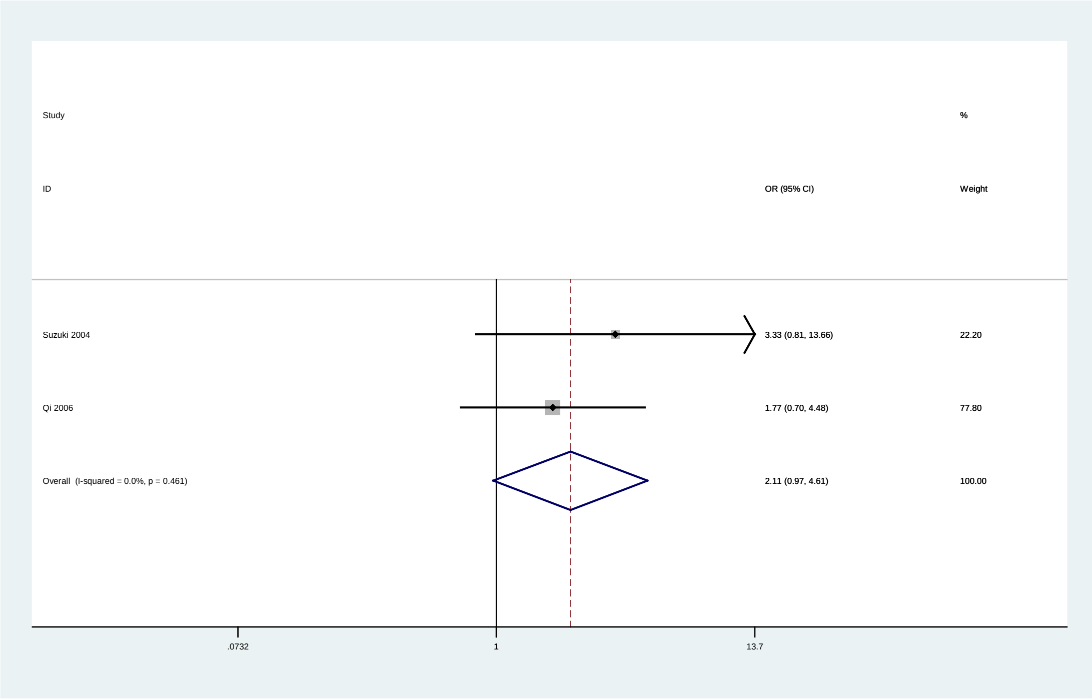

Figure S2: Forest plot for the association of methylated *SFRP4* between 87 CRC patients and 63 benign mucosal lesions, OR = 2.11, 95% CI = 0.97 - 4.61, P = 0.06. Abbreviations: CI, confidence interval; OR, odds ratio; CRC, colorectal cancer.

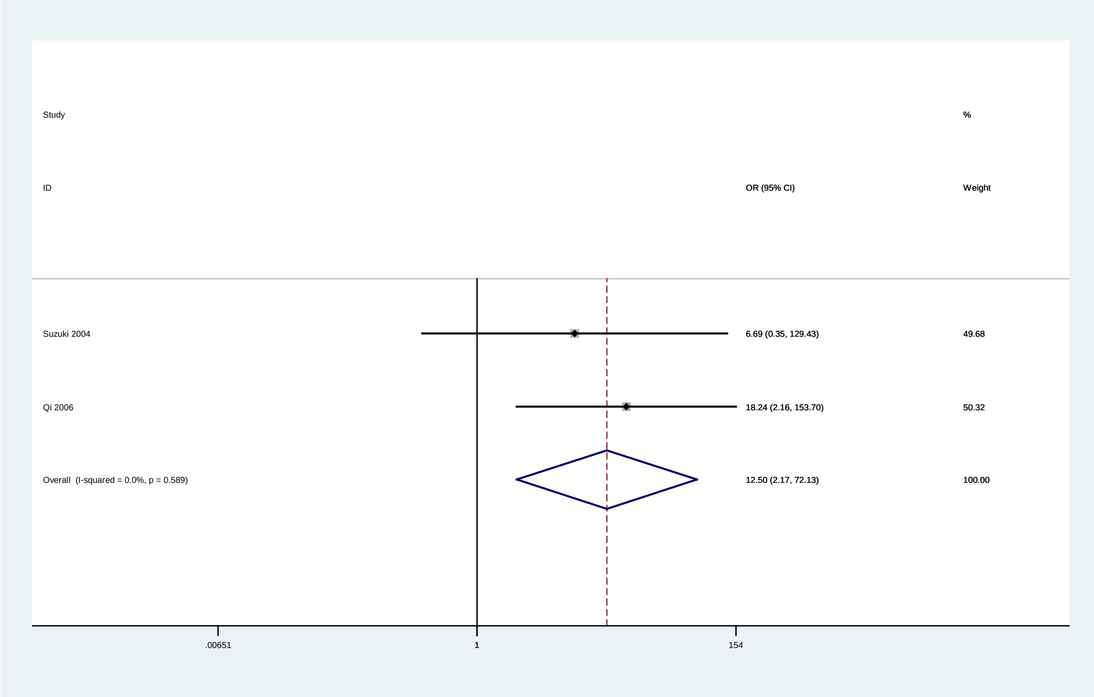

Figure S3: Forest plot for the association of methylated *SFRP4* between the 63 benign mucosal lesions and 73 normal colonic mucosa, OR = 12.50, 95% CI = 2.17 - 72.13, P =0.005. Abbreviations: CI, confidence interval; OR, odds ratio; CRC, colorectal cancer.

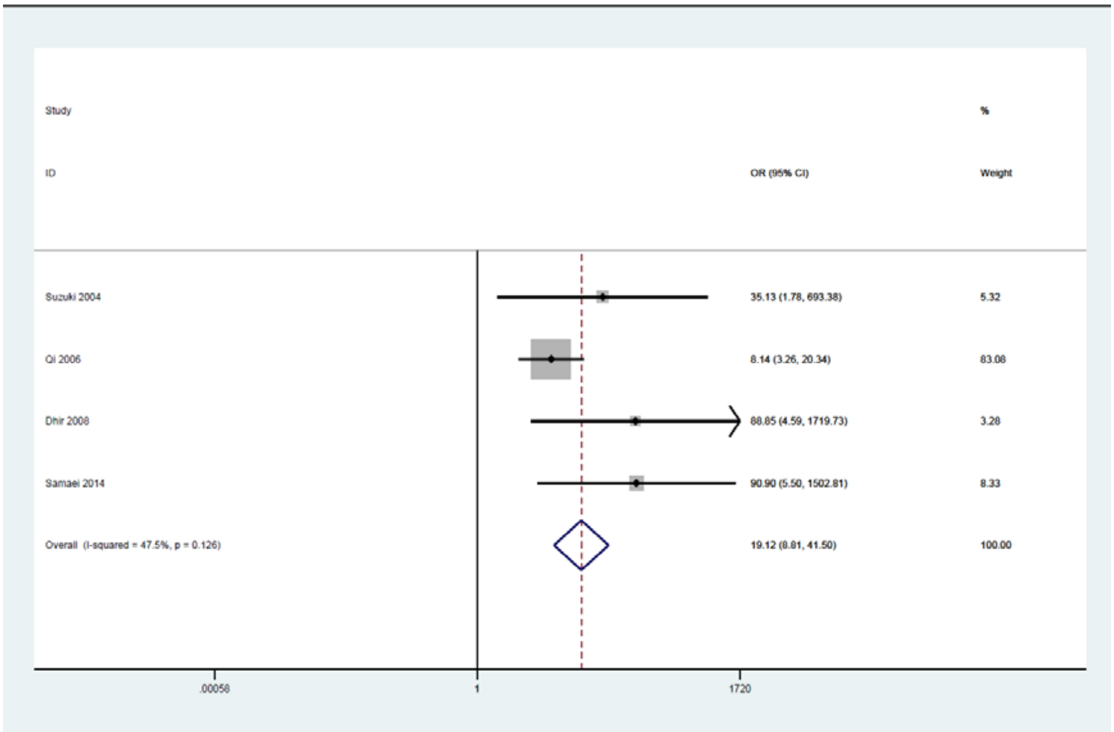

Figure S4: Forest plot for the association of methylated *SFRP5* between 228 CRC patients and 225 normal colonic mucosa, OR = 19.12, 95% CI = 8.81 - 41.50, P < 0.001. Abbreviations: CI, confidence interval; OR, odds ratio; CRC, colorectal cancer.

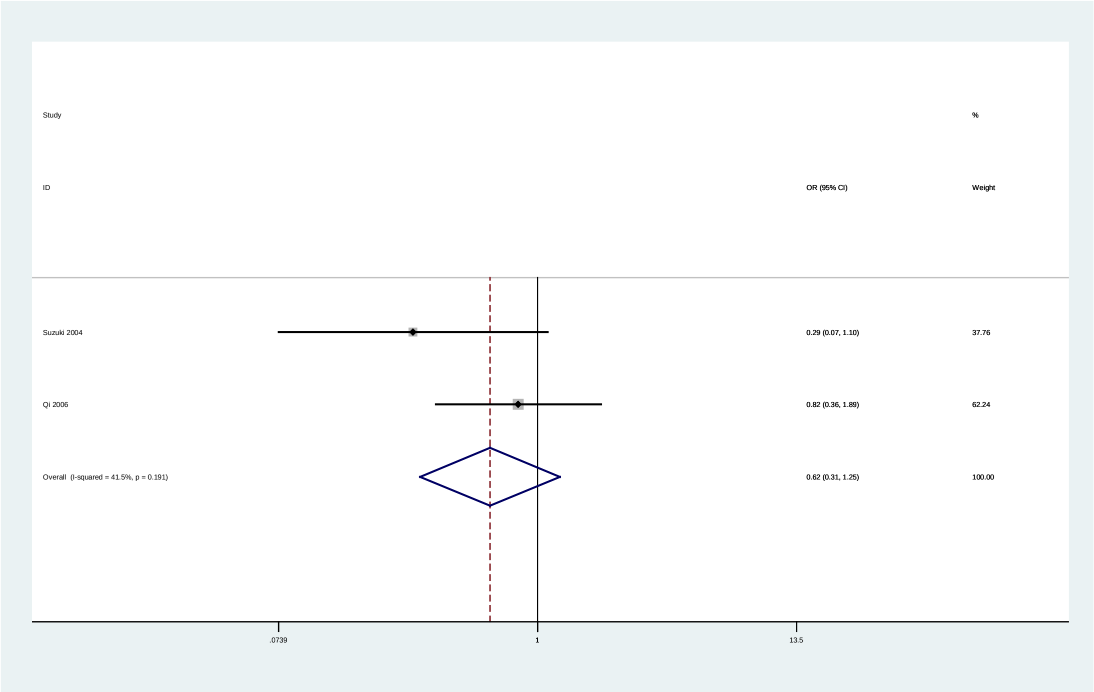

Figure S5: Forest plot for the association of methylated *SFRP5* between 87 CRC patients and 63 benign mucosal lesions, OR = 0.62, 95% CI = 0.31 - 1.25, P = 0.183. Abbreviations: CI, confidence interval; OR, odds ratio; CRC, colorectal cancer.

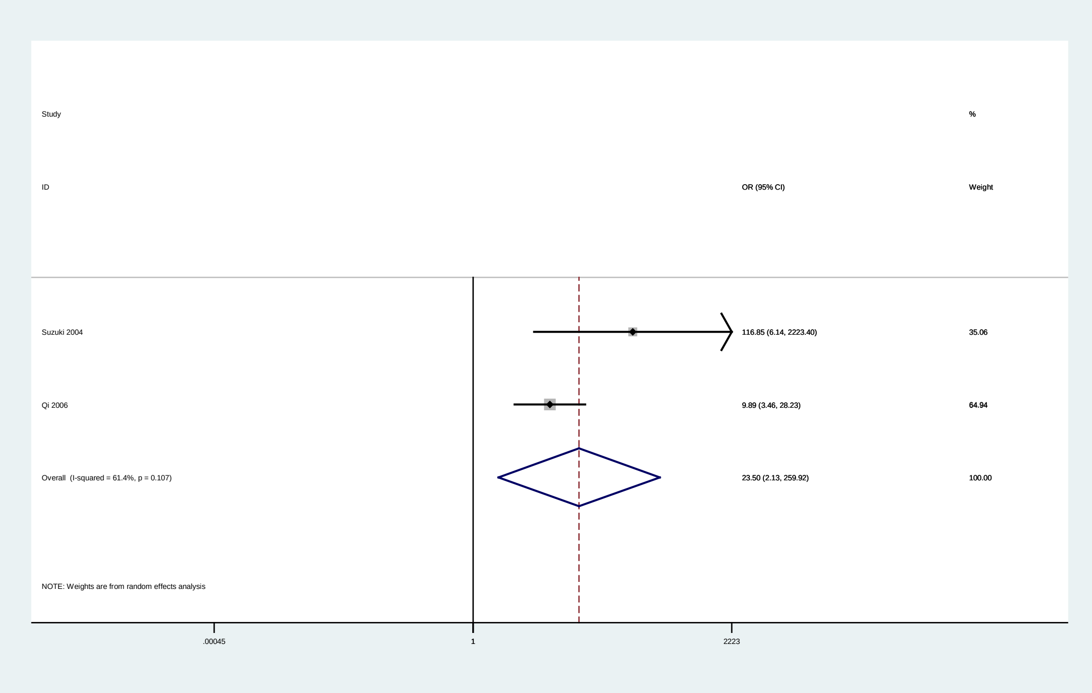

Figure S6: Forest plot for the association of methylated *SFRP5* between 63 benign mucosal lesions and 73 normal colonic mucosa, OR = 23.50, 95% CI = 2.13 - 259.92, P = 0.01. Abbreviations: CI, confidence interval; OR, odds ratio; CRC, colorectal cancer.
